# Abscesses of the Ano-Perineal Region

**Published:** 1883-05

**Authors:** Philip S. Wales

**Affiliations:** Surgeon General U. S. Navy


### Independent Practitioner.

VOL. IV. MAY, 1883. No. 5.

### Original Communications.

We are not responsible for the opinions expressed by contributors.

### ABSCESSES OF THE ANO-PERINEAL REGION.

BY PHILIP S. WALES, SURGEON GENERAL U. S. NAVY.

### CLASSIFICATION.

In the classification of abscesses occurring in the neighborhood of the anus, either the clinical or the anatomical scheme may be followed, though the former is usually adopted by systematic writers. Gosselin divides anal abscesses into four classes, according to their symptoms and course ; while Chassaignac describes no less than seven varieties : three superficial—the tuberose, the phlegmonous, and the circumscribed phlebitic ; and four deep—that occurring in the connective tissue of the ischio-rectal and the superior pelvi-rectal spaces, that connected with lesions of the rectum, that associated with diseases of the urinary organs, and the osteopathic. An anatomical classification would group abscesses according to their seat, whether in the skin and subjacent cellular tissue, in the glandular appendages, in the connective tissues of the ischio-rectal and superior pelvi-rectal spaces, in the tissues of the rectal walls, or in the tissues of dilated veins. The division which will be selected for convenience of description, is that which groups them in two great clinical classes; the superficial and the deep.

SUPERFICIAL ABSCESSES.

A *common form* of abscess occurring about the anal aperture is that which is located in the secretory appendages of the skin—the sudoriferous and sebaceous glands—and which is analogous in nature to the tarsal and vulvar phlegmons arising in the corresponding appendages of the skin of those regions. In certain cases it shows a persistent tendency to recurrence; male subjects are more prone to it than female, and persons in apparently robust health than the feeble and sickly; it is rarely encountered in infancy or old age.

*Symptoms.*—The disease begins with tenderness and pain in the perineal region, which is greatly increased in defecation and in sitting. At some point near the anus a blush of redness with a hardness is observed, which later, forms a distinct circumscribed swelling about the size of a filbert. This stage endures four or five days, when softening begins with the formation of pus, which is finally discharged with marked alleviation of the symptoms. The pus is small in quantity, and almost always fetid; it becomes thin and serous, and continues to drip from the orifice a few days. In the meantime the hardness of the part disperses, and the pus cavity contracts; then one of two things happens; either the orifice definitely closes by cicatrization, or a sinus is left which continues to secrete indefinitely, forming a fistula.

*Etiology.*—The disease arises from various sources of irritation; the contact of perspiration chemically altered by the heat of the locality, assisted by the mechanical effects of friction between the opposing surfaces of the gluteal muscles; the rubbing of the parts by the seams of badly made or ill fitting garments; neglect of proper attention to habits of cleanliness; injuries from blows, falls, hard riding, etc.; the passage of masses of hardened feces bruising the sphincter; or the irritating effects of acrid discharges from the rectum or the vagina.

*Treatment.*—The treatment best adapted to this form of abscess is early incision. I am in the habit of doing this as soon as the part becomes hard and painful. The incision disgorges the tissues and offers the only hope of aborting the inflammation ; an additional merit is the fact that many hours of suffering are eliminated by relieving the tension of the tissues. If later incision should be deemed advisable, the best course to pursue is to make local applications of flannel or sponge, wrung out of hot water and dashed with tincture of opium or other sedative. There are those who prefer poultices, made of bread crumbs, flax-seed meal, or scraped Irish potato.

Still more frequent than the preceding form is the *phlegmonous abcess,* which has its origin in the subcutaneous connective tissue. It may occupy any position in the perineum, though usually it is found from one to three centimeters from the anus, on one or the other side—rarely in front or behind—and it is more diffused than the former. Seemingly healthy persons are often the victims of the disease, yet it does not spare those whose health has been impaired by other maladies. The middle period of life furnishes the greatest number of examples, though one case, a child three years of age, came under my observation. Women are less liable to it than men.

*Symptoms.*—It begins with pain and swelling of the part, and more or less induration and œdema of the surface, which is smooth, red and shining, or of a dusky or purplish hue. The pain is aggravated by pressure with the fingers, or in sitting, as well as by the concussion produced by sneezing and coughing ; defecation is always painful, and occasionally exquisitely so. There is slight constitutional disturbance, the patient feels ill and feverish, has anorexia, and is disinclined to move about. After five or six days the induration decreases, and fluctuation may be detected at some one point, where, finally, the skin breaks and a quantity of pus escapes with marked amelioration of the sufferings. The purulent matter continues to flow for some days—a week or more—and as the process of absorption progresses the infiltrated tissues are relieved of their burthen and assume their normal softness and elasticity, by which time the aperture is definitely closed. This favorable termination is, unfortunately, not always reached ; in certain cases, in consequence of the peculiarity in the local condition and function of the part, or from some indefinable constitutional cause, the process of repair stops short, leaving a canal or sinus, walled about by indurated tissue and secreting a serous fluid for an indefinite time.

*Etiology.*—The exciting causes of phlegmonous abscess are traumatic influences acting from without, or from within; among the former are included injuries from blows and falls, and surgical operation for hemorrhoids, fissure, and fistula. The interior causes spring from the lodgment in some point of the rectal walls of foreign bodies that have been swallowed, where perforation into the circumjacent connective tissue finally occurs. The abscess is sometimes found linked with other diseases, either of a constitutional or local character; thus, it may complicate the convalescence from the various forms of low fever, or tuberculosis; or it may be coupled with affections of the neighboring organs, as with gonorrheal inflammation of the vesiculæ seminales, as Gosselin has asserted, and which he regards as furnishing an explanation of the frequency of perineal abscess in the male.

*Treatment.*—The practice I have followed in phlegmonous abscess, is to incise the indurated part at the more prominent point, or at that point which would seem to be the one where the pus would most likely point, and then to apply hot fomentations. Even when no pus is discharged by the incision the patient experiences the most signal relief to his sufferings by the bleeding, and consequent removal of tension of the part. I have certainly seen the extension of the inflammation checked, if not aborted by this simple operation. The incision need not be ordinarily more than a centimeter in length, and two or three deep, and the hot fomentations may be continued to relieve pain and to facilitate the dispersion of the induration. It has been recommended that efforts should be made to abort the inflammation by the application of ice, but I can not recall successful examples of this mode of treatment, within the sphere of my observation.

*Another Form* of abscess is that originating in a segment of an enlarged hemorrhoidal vein, which has been cut off from the venous circuit by inflammatory changes in its walls. Under these circumstances the obstructed vein is usually converted into a small, solid, indolent tumor. It sometimes happens, however, that the irritation is sustained by the persistence of various local causes, as the contact of acrid secretions, or the bruising caused by the contact of hardened feces, and suppuration is induced. The vein is then converted into an elastic, painful, smooth, and rounded tumor, which rarely attains a larger size than a small marble. It may be located at the margin of the anus, or within the sphincter, and occasions much distress and pain during defecation. The treatment required is timely incision, and the local use of hot water holding anodyne remedies in solution.

DEEP ABSCESSES.

These abscesses differ from the preceding class in that they are formed in the deeper structures of the perineum, and are, therefore, of a much more serious character. Their habitat is the celluloadipose tissue of the great space at the outlet of the pelvis, which is bounded above by the under surface of the peritoneum, as it is reflected from the pelvic wall to the rectum and bladder; internally, by the rectum; externally, by the ischium and obturator internus muscle; posteriorly, by the sacrum; and below by the skin and subjacent connective tissue. This region is divided in two by the levator ani muscle; the upper part, Richet has designated as the superior pelvi-rectal space, while the lower part is that which has long been known as the ischio-rectal fossa. Although anatomically the locality of the abscess is different, as regards the levator muscle, yet its course is the same, except that the suppurative action at the higher point is nearer the peritoneum, and, therefore, liable to spread more widely in the connective tissue to contiguous parts, or even invade the peritoneal cavity; and, the purulent matter is longer in reaching the surface, through the musculo-membranous septum formed by the levator. For these reasons it is more dangerous.

The disease has long been known to surgeons under the various titles of phlegmonous, stercoral, gangrenous, and ischio-rectal abscess. Chassaignac divides deep abscesses in four groups, according to their etiological origin : those originating directly in the cellulo-adipose tissue of the ischio-rectal fossa ; those which recognize rectitis as a cause from whatever source this may spring —traumatic, ulcerous, phlebitic, or parenchymatous ; those resulting from some lesion of the genito-urinary organs ; and lastly, those that are the direct result of disease of the bony walls of the pelvic cavity.

The anatomical characters of deep abscesses vary in different cases. The collection of purulent matter may occupy a single cavity in the ischio-rectal fossa, and communicate with the exterior either through the rectum, or at the perineum. In some cases, on the other hand, there is a second cavity, formed beneath the mucous tunic, which is dissected up to a greater or less extent, and this cavity is connected with that in the fossa by a narrow aperture in the muscular wall of the bowel, thus forming together an hour-glass shaped abscess—the *abcès en bissac* of the French writers. A similar conformation is also observed when the pus forms in the superior pelvi-rectal space, and makes its way through the levator ani muscle by a narrow aperture into the ischio-rectal fossa. Again, an abscess may occupy each ischio-rectal fossa, and they may communicate by a channel located near the tip of the coccyx and beneath the sphincter ani. From the peculiarities of structure and function of the perineum—the constant mobility of its muscular constituents during the act of defecation and respiration, and the changing volume of the neighboring organs from repletion or emptiness—a state of continual unrest results, which influences the course of pathological processes occurring in this region, and hence the difference in the course of abscess here as compared with that of other parts of the body. Even when the purulent matter has been freely evacuated the walls of the cavity do not fall in contact, but remain separated for the reasons above stated, and great delay necessarily ensues in the completion of the reparative process. Further difficulties are encountered in the drainage of pus through the narrow and devious channels that run to the exterior, and also in the hindrance to its flow presented by the narrow orifices in the muscular layers through which these channels course.

*Etiology.*—Traumatic influences hold the first rank in the etiology of deep perineal abscesses. They may be exercised from without or from within. The external causes are falls and blows upon the perineum ; the wounds inflicted by pointed instruments as sticks, pronged tools, etc. I saw a case of abscess in a boy, which resulted from falling upon the prongs of a hay-fork. It sometimes happens that wounds inflicted in the performance of surgical operations lead to the same result, as in the removal of hemorrhoidal tumors by ligature or knife; it may occur from the rough introduction of the clyster pipe, causing a rupture of the mucous membrane, thereby opening the connective tissue spaces to the irritating fluid employed ; lastly, may be mentioned the wounds inflicted by the forcible introduction into the anus of foreign bodies, either accidentally or designedly. The internal sources of traumatism include the lodgment in the rectum of foreign bodies that have been swallowed, and which subsequently perforate it, and lead to extravasation of fecal matter into the connective tissue ; or hardened feces or enteroliths. The passage of the fœtal head has in some cases bruised the rectum, and excited suppuration in the ischio-rectal fossa. Inflammation of the sub-mucous veins may lead to abscess which dissects the inner from the muscular coat, and finally invades the peri-proctal tissues, or bursts into the rectum and thus opens up the way for fecal extravasation. Strictures which obstruct the passage of fecal matter often give rise to extensive dilatation of the gut and subsequent ulceration and extravasation of the intestinal contents. Disease of the adjoining organs may be the starting point of the suppuration, as occurs sometimes in inflammation of the prostate, bladder and urethra. A case came under my notice of a man of intemperate habits, one who had long suffered from gleet, and was seized with prostatitis, after a debauch in which he was exposed to cold, which ran on to the formation of an abscess. The matter made its way into the perineum, and at the same time broke into the urethra, but there was no infiltration of urine in this case, and the man made a safe and speedy recovery. Rupture of the urethra, and extravasation of urine from stricture furnishes some of the most aggravated cases. An example of this sort came under my care during the late war. A metal sound intended to dilate an impassable stricture was thrust out at the perineum near the anus, and caused extravasation of urine and abscess. Necrosis of the bony walls of the pelvis is the source of the disease in rare cases. Exposure to cold, or sitting on the damp ground, furnish a small contingent. There are other cases in which the most careful scrutiny fails to discover any cause to which the disease might be attributed.

*Symptoms.*—In ischio-rectal abscess the initial phenomena are slight; there is a sense of weight or dull pain in the ano-perineal region, the surface after a time becomes red, hot and œdematous, the parts are tender under manipulation; distress is felt in the sitting posture, and the patient may be a little feverish. Although rigors and an increase of fever may indicate the formation of pus, yet the closest examination often fails in determining any point of softness or fluctuation in the perineum; the parts are everywhere hard and resisting. The suffering, however, becomes by degrees more intense, and continues so until the pus makes an exit at some point. This may occur in the rectum, when the event will be indicated by the bursting of the abscess at stool, and the escape of matter from the anus; in rare cases it takes an outward course, and escapes at the sciatic foramen upon the buttocks, or, as is most often the case, downwards into the perineum or upon the thigh, as in the following example:

A man, æt 47, came under my care with this history: "In Sept., 1860, an abscess occurred near the left tuberosity of the ischium, extending towards the perineum, a sinus extending upon the posterior part of the thigh, and one upon its inner side. He complained of pain about the left hip, especially in walking. There was also a persistent cough." In December I saw him. I found that the sinuses of the buttocks and thigh were connected with a perineal abscess, into which I made an incision and established free drainage ; the cavity contracted slowly, and the following February the patient had regained his health.

In other instances, it bursts through the levator ani, and gaining admission into the tissue of the superior pelvi-rectal space it follows this muscle along to the groin, where the pus points, or it may pass beneath Poupart's ligament and reach a lower opening; in exceptional cases, the pus may break into the peritoneum. Should the pus invade the interval between the bladder and rectum, the vesical functions will be more or less embarrassed, and retention of urine, or other trouble may follow. When the abscess starts in the peri-proctal tissues, the finger introduced in the rectum will, in the early stage, indicate its position by the induration of the rectal walls, or later, by fluctuation and the protrusion of the tumor into the lumen of the gut. It sometimes happens that this induration presents itself and persists without being followed by suppurative action, as was the case in a patient of mine, a lady who had been run down in health by certain physical causes and mental distress. The symptoms of perineal abscess began to show themselves, and I detected an induration in the periproctal tissue ; hot water injections were employed with assiduity. No pus formed, but the hardness persisted for a long time with moderate distress in defecation. Under tonic treatment and change of air the patient fully recovered. In exceedingly rare cases, the symptoms of ileus are said to have been observed, as recorded by Boens.

In cases that are, fortunately, rare, the violence of the inflammation induces gangrene of the perineal structures and even of the rectum itself. This result is indicated by the skin assuming a deep red or purplish color, the cuticle is elevated into vesicles containing bloody serum, and finally soft, yellow or dark sloughs of larger or smaller dimensions are thrown off, leaving ragged chasms in the perineum, discharging offensive sanies. The breaches in the part sometimes occupy the whole of one ischio-rectal fossa, and form a sort of diverticulum to the rectum, in which fecal matter may collect, and still further complicate the case.

The following case, taken from my note book, is one of six of a similar character, which illustrates these evil results, and at the same time, shows how speedily amendment occurs after careful evacuation and drainage at a late period in the case. A sailor, aged 45, came under my care in March, 1859. He had had an abscess of the right ischio-rectal fossa. The parts had been much tumefied and exceedingly painful. There were three small orifices observed, discharging thin, fetid, and bloody pus. The case had been persistently treated with poultices, with the result that the cellulo-adipose tissue finally sloughed *en masse*, leaving a large, deep, cavernous excavation, at the bottom of which sinuses ran upwards in several directions; the probe could be passed up the side of the rectum more than three inches and the gut itself was dissected from its connections. With the finger and handle of the knife I enlarged the wound, and succeeded in opening a pocket high up, which discharged a quantity of pus of exceedingly fetid odor, with immediate relief to the agonies which the patient was suffering. Considerable hemorrhage followed, but was soon checked by packing the wound with lint. This dressing was removed next day, and the cavity lightly filled with charpie soaked in a solution of chlorinated soda, so that the discharge had free issue. This line of treatment was continued until the cavity filled up, and in April the parts were completely healed and the man resumed his duties.

Abscess of the *superior pelvi-rectal space* causes a sense of weight and fulness in the perineum, which finally becomes very painful while the pus is making its way to the surface. If this happens to occur at the perineum, the parts present the indurated appearances described in the former case; if at the inner surface of the rectum, there will be a discharge of purulent matter from the anus, especially during defecation. When the position of the inner orifice is located high, the pus will be deposited upon the fecal discharge, while on the contrary if low, the matter will be extruded first, and thus occupy a position beneath the fecal discharge. The finger introduced into the rectum will detect the indurated tissue, and the exercise of pressure will cause the matter to issue from an external orifice in considerable quantity, as long as there is any in the pocket above the levator ani. When there is a perineal orifice, the probe penetrates deeply, if the sinuosities of the fistulous conduit can be surmounted by that instrument, and the usually thick stratum of tissue intervening between it and the rectal wall can be felt by the finger.

*Prognosis.*—Deep perineal abscess is always serious, and often dangerous, especially that rare form which depends upon necrotic conditions of the pelvic bones and of the vertebræ. When abandoned to itself, the abscess may become the focus of septic infection, phlebitis, or embolic abscesses of the liver. The constant purulent drain exhausts the vital powers, and may lead to various secondary complications, as amyloid degeneration,or tuberculosis, not to mention the lesser evils of permanent fistula, incontinence of feces from the destruction of the integrity of the sphincters,stricture and other secondary effects of contracting cicatricial tissue upon the vagina and urethra. The parts remain long tender and susceptible to slight irritating causes.

*Treatment.*—Little if any difference of opinion is now entertained as to the proper mode of treatment of deep perineal abscess. There may exist some divergence in practice as to the details, regarding the length, depth and form of the incision, yet it is universally admitted that the early use of the knife is indispensable. It is rarely possible to abort the disease when it has once begun, by the resolvent measures usually recommended for this purpose. Their employment simply consumes valuable time in which the knife alone can accomplish the arrest of the destructive ravages of diffuse inflammation of the connective tissue of the pelvis, and the denudation of its organs. Anatomical considerations explain these results of delayed incision ; the perineal fascia, closing in the outlet of the pelvis with a resisting floor, oppose the escape of matter situated above it, and thus turn its course into the more yielding connective tissue spaces.

Perfect evacuation of pus and subsequent thorough drainage are the indications to be fulfilled, and all surgical procedures should be directed to secure these objects, and with the least damage to the muscular structure of the anus and rectum. In those cases which fall under the notice of the surgeon in their incipiency, a single antero-posterior linear incision of sufficient length and depth will suffice ; but if the pus has made its way towards the thigh, or buttock, it may be necessary to extend the incision laterally by a second incision at right angles to the first. It should then be determined whether any adjacent pockets or sinuses containing matter are present, and if so they should be made to communicate freely with the wound by the use of the knife or finger. The next step is to introduce carbolised rubber tubes into the deepest recesses of the wound, to fill the space around the tubes with antiseptic cotton, and to retain the whole by an appropriate bandage. As the pus drains away the cavity of the abscess contracts ; even though the rectum may have been dissected from its connections, the breach of continuity of tissue may be repaired without fistulous tracks being left. The same method of treatment should be followed, when the abscess has already broken through the rectal walls and established fistulous communications, for these may spontaneously close as the healing of the abscess progresses ; should this result not occur when the reparation of the parts have otherwise been completed, recourse may then be had to the ordinary operation for fistula. It is not necessary to follow all the devious windings leading from the abscess into the neighboring regions, as they are likely to heal spontaneously with the closure of the purulent cavity.

